# *Malus xiaojinensis MxbHLH30* Confers Iron Homeostasis Under Iron Deficiency in *Arabidopsis*

**DOI:** 10.3390/ijms26010368

**Published:** 2025-01-03

**Authors:** Yu Xu, Yingnan Li, Zhuo Chen, Xinze Chen, Xingguo Li, Wenhui Li, Longfeng Li, Qiqi Li, Zihan Geng, Saiyu Shi, Lihua Zhang, Deguo Han

**Affiliations:** Key Laboratory of Biology and Genetic Improvement of Horticultural Crops (Northeast Region), Ministry of Agriculture and Rural Affairs, National-Local Joint Engineering Research Center for Development and Utilization of Small Fruits in Cold Regions, College of Horticulture & Landscape Architecture, Northeast Agricultural University, Harbin 150030, China; xuyu37822@163.com (Y.X.); liyingnan1031@163.com (Y.L.); a02220019@neau.edu.cn (Z.C.); a02220107@neau.edu.cn (X.C.); xingguoli@neau.edu.cn (X.L.); wenhuili@neau.edu.cn (W.L.); 18263040626@163.com (L.L.); 15966263026@163.com (Q.L.); 19861731368@163.com (Z.G.); ssy2864321318@163.com (S.S.)

**Keywords:** *MxbHLH30*, *Malus xiaojinensis*, Fe stress, gene transformation

## Abstract

Iron stress adversely impacts plants’ growth and development. Transcription factors (TFs) receive stress signals and modulate plant tolerance by influencing the expression of related functional genes. In the present study, we investigated the role of an apple bHLH transcription factor *MxbHLH30* in the tolerance to iron stresses. The expression of *MxbHLH30* was induced significantly by low-iron and high-iron treatments and *MxbHLH30*-overexpressed *Arabidopsis* plants displayed iron-stress-tolerant phenotypes. A determination of physiological and biochemical indexes associated with abiotic stress responses showed that overexpression of *MxbHLH30* increased the activities of antioxidant enzymes superoxide dismutase (SOD), peroxidase (POD) and catalase (CAT) in *Arabidopsis* plants treated with iron stress, and decreased the contents of H_2_O_2_ and malondialdehyde (MDA), which contribute to reduce cell membrane lipid peroxidation. Meanwhile, the accumulation of proline in transgenic plant cells increased, regulating cell osmotic pressure. Furthermore, quantitative expression analysis indicated that overexpression of *MxbHLH30* improved the expression levels of positive functional genes’ responses to iron stress, improving plant resistance. Interestingly, *MxbHLH30* may have the ability to balance the homeostasis of iron and other metal ions for the iron homeostasis of *Arabidopsis* cell under low-iron environments. This research demonstrates that *MxbHLH30* is a key regulator of cell iron homeostasis in *Arabidopsis* plants under iron deficiency, providing new knowledge for plant resistance regulation.

## 1. Introduction

Environmental conditions that are unfavorable to plant growth, such as salinity, iron deficiency, high temperatures, cold and drought, have a significant impact on the production and quality of plants [1,2,3]. Plants first detect stress and recognize pertinent information through a signal transduction network in response to biotic or abiotic stressors [4]. Eventually, stress-responsive genes are transcribed, causing changes in physiological processes and further transduction of stress signals [5]. The morphological adaptations and in vivo changes in physiological metabolism that occur in plants in response to adverse external environments are produced by a series of gene regulation processes. These genes include signaling cascade genes, regulatory genes, functional genes and other related genes [6]. In response to abiotic stresses, the network signaling cascade response is achieved through numerous transcription factors (TFs) [7], one of which is the bHLH transcription factor.

A conserved bHLH functional domain with a basic region and a helix–loop–helix (HLH) region is present in the bHLH transcription factor [8]. The ability to bind to downstream genes that are linked depends on these two regions [1,9,10]. With the completion of genome sequencing of more species, numerous plant bHLH proteins, such as in sweet potato [11], *Arabidopsis thaliana* [12], sweet cherry [13] and grape [14], respectively, are being discovered and studied. Studies have shown that bHLH TFs are important for the regulation of plant stress resistance. Many members of the family have direct roles in the plant stress response process. They control transcriptional regulation and post-translational modifications to control how the plant adapts to external challenges [15].

Medium mineral elements like Fe, Zn, Mn, Cu and others are equally important to plant growth and development. For the development of plants and their growth, the micronutrient iron (Fe), which is also a part of many plant proteins, is essential. Via the transfer of electrons, iron participates in redox reactions in plants and is involved in a number of biological activities, including the creation of hormones, photosynthesis, chlorophyll and mitochondrial respiration [16,17]. However, a variety of factors, including interactions between various nutrients, the pH of inter-root soil and variations in hormone concentrations in plants, affect the uptake and utilization of Fe by plants. Therefore, iron deficiency due to the accumulation of insoluble Fe and Fe (III) needs special attention.

In terms of interactions between various trace elements, the antagonistic relationship between Fe and Zn has been studied in plants [18,19]. Zn binds more strongly to transporter proteins than Fe, resulting in an easier displacement of Fe. The uptake and buildup of Mn and Zn are accelerated by iron deficiency [20]. Zn toxicity is frequently exacerbated by iron insufficiency, while Zn toxicity can be lessened by raising Fe levels [19,21]. Iron-mediated Zn tolerance may be maintained by iron-regulating metal transporters to prevent high Zn uptake in *Arabidopsis* [21,22]. In addition, Zn uptake also contributes to plants exhibiting a stronger Fe deficiency response [23,24]. Lešková et al. [25] demonstrated that excessive Zn application mimics the yellowing of plants caused by iron deficiency, leading to a decrease in chlorophyll concentration and an increase in ferric chelate reductase activity. These reactions were attributed to Zn mimicking the transcriptional responses of iron-regulated key genes [25]. In conclusion, Zn availability influences Fe absorption and homeostasis in plants, and vice versa.

*Malus xiaojinensis* is an iron-efficient genotyped apple germplasm resource. In recent years, numerous genes associated with iron stress have been discovered, and extensive research has been carried out on the molecular processes of iron uptake and transport in *Malus xiaojinensis* [26]. *MxMYB1* and *MxERF4* may act as negative regulators of iron uptake and storage [27,28]. High *MxNAS1/2/3* expression levels strengthened tolerance to high- and low-iron stress, but they also caused transgenic tobacco to blossom later and with aberrant flowering [29]. MxbHLH01 may regulate iron homeostasis by forming heterodimers with other proteins [30]. Phosphorylation of MxbHLH104 by MxMPK6-2 in vivo increases Fe uptake by apple healing tissues [31]. However, it is unclear how the bHLH gene affects *Malus xiaojinensis*’s stress response. In this study, significant variations in the expression of several bHLH transcription factors were discovered by comparing the transcriptome data of *Malus xiaojinensis* produced by iron stress. Thus, we further screened these genes by qRT-PCR and chose *MxbHLH30*, which was significantly induced by stress and remained at a higher expression level from 3 to 9 h after stress, as a candidate gene. The function of *MxbHLH30* was analyzed and characterized to provide a new candidate gene for breeding for iron deficiency, as well as to provide the basis for deep analysis of the stress response mechanism of *Malus xiaojinensis*.

## 2. Results

### 2.1. Isolation and Phylogenetic Relationship of MxbHLH30

**Figure 1:**
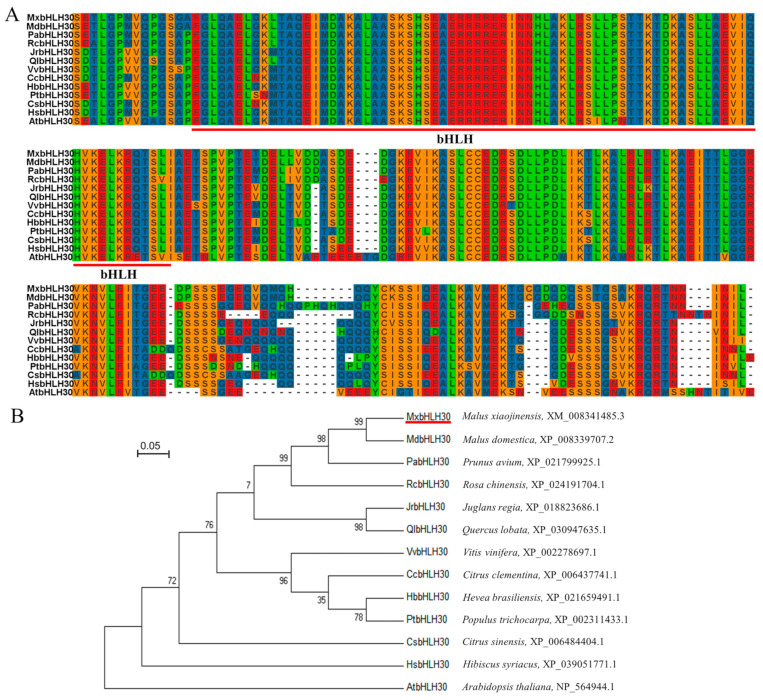
Evolutionary relationships and subcellular localization analysis of MxbHLH30. (**A**) Sequence alignment of MxbHLH30. Amino acid sequences shown in the red underline are bHLH-conserved structural domains. (**B**) Evolutionary tree analysis of MxbHLH30 and other species.

The full-length coding region of *MxbHLH30* was separated from *Malus xiaojinensis*. MxbHLH30 encodes 349 amino acids (Figure S1) and 10.9% Leu (L), 9.2% Gln (Q), 9.2% Ser (S) and 7.2% Glu (E), with the molecular weight (MW) of 38.943 kDa, the theoretical isoelectric point (pI) of 6.15 and the average hydrophilicity coefficient of −0.645 (Figure 1A).

The *MxbHLH30* (XM_008341485.3) and its homologous genes were obtained from the Apple Genome Database (https://www.rosaceae.org, accessed on 7 March 2023) and National Center for Biotechnology Information (NCBI) Database, and the conserved amino acid sequences of all tested *bHLH* genes are depicted in Figure 1A. Phylogenetic analysis of MxbHLH30 and its homologous proteins from 12 other species indicated that MxbHLH30 possessed high homology with MdbHLH30 (*Malus domestica*, XP_008339707.2), followed by PabHLH30 (*Populus alba*, XP_021799925.1) and RcbHLH30 (*Rosa chinensis*, XP_024191704.1), which form the first cluster of the evolutionary tree (Figure 1B).

### 2.2. MxbHLH30 Protein Was Localized in the Nucleus

**Figure 2:**
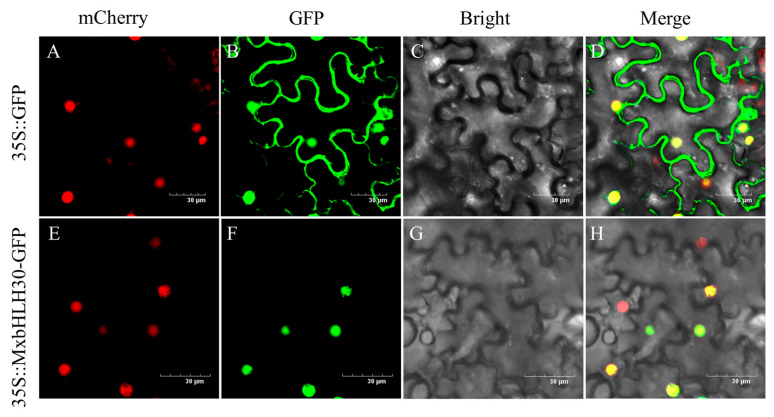
Subcellular localization of MxbHLH30 protein. 35Spro::MxbHLH30::GFP was expressed transiently into tobacco leaves with 35Spro::GFP as positive control. (**A**,**E**) mCherry; (**B**,**F**) GFP signals; (**C**,**G**) bright field; (**D**,**H**) merge. mCherry as a nuclear marker. Yellow indicates GFP and mCherry colocalization. Scale bars correspond to 30 µm.

To analyze the subcellular localization of the MxbHLH30 protein, a 35S::MxbHLH30::GFP fusion expression vector was constructed and transformed into tobacco leaves with 35S::GFP as a control. Green fluorescent protein (GFP) of the control was seen in the membrane and nucleus of the cell, whereas 35S::MxbHLH30::GFP was only localized to the nucleus under fluorescence confocal microscopy (Figure 2). In addition, MxbHLH30 could be identified as a nuclear-localized protein by observing the red fluorescence emitted from the nucleus.

### 2.3. Expression Analysis of MxbHLH30 in Malus xiaojinensis

**Figure 3:**
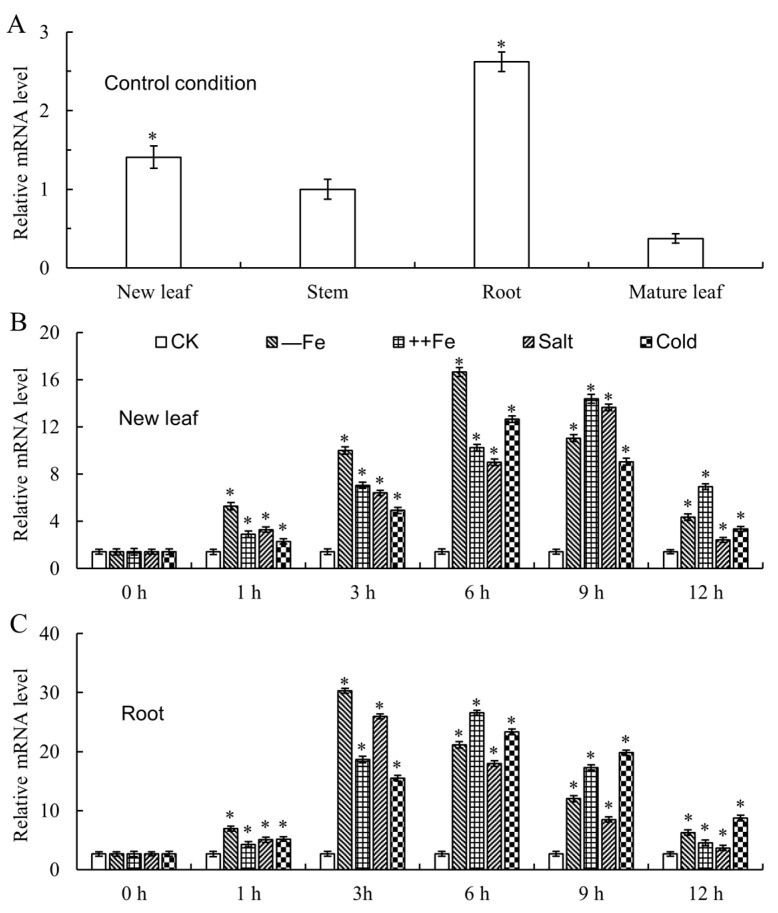
Expression patterns of the *MxbHLH30* gene in several tissues of *Malus xiaojinensis* induced by adversity stress. (**A**) The expression level of *MxbHLH30* in three organizations of *Malus xiaojinensis* grown in a normal environment (100 µM Fe-EDTA). Four stimuli, including low iron (—Fe), high iron (++Fe), high salt (200 mM NaCl) and low temperature (4 °C), caused the expression of *MxbHLH30* genes in new leaves (**B**) and roots (**C**). The average and standard deviation of three replicates were used as the data. Asterisks on the bars denotes a difference from the control that is significant (* *p* ≤ 0.05).

To analyze the expression patterns of *MxbHLH30* in various tissues of *Malus xiaojinensis,* quantitative real-time polymerase chain reaction (qRT-PCR) was performed to evaluate its expression in new leaves, stems, roots and mature leaves. The results illustrated that *MxbHLH30* showed the highest expression levels in root and new leaves, while relatively low levels in mature leaves and stems (Figure 3A).

Subsequently, the expressions of *MxbHLH30* under low-iron, high-iron, salt and low-temperature stress treatments also were analyzed. As shown in Figure 3B, the expression of *MxbHLH30* exhibited a rising and subsequently dropping trend within 12 h in four stress conditions. In detail, *MxbHLH30* expression peaked at 3 h, which was up to 10-fold higher than that of the control in roots treated with low iron. A similar phenomenon was also observed after 6 h with high-iron treatment. In keeping with the variation trend of *MxbHLH30* expression in treated roots, the expression in leaves under various stresses was also enhanced and then declined (Figure 3C). It is noteworthy that iron more strongly induced the expression of *MxbHLH30* compared with low temperature and salt, suggesting that *MxbHLH30* is extremely sensitive to iron stress.

### 2.4. Overexpression of MxbHLH30 in Arabidopsis Enhances High- and/or Low-Iron-Stress Tolerance

**Figure 4:**
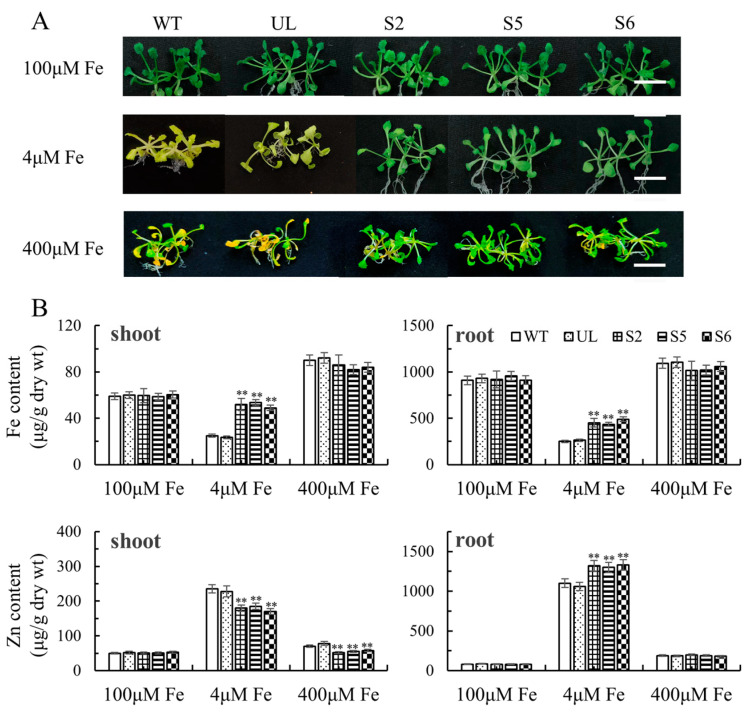
Fe stress tolerance is developed by *MxbHLH30* overexpression in *Arabidopsis*. (**A**) S2, S5, S6, WT and UL phenotype under low iron (4 µM Fe-EDTA) and high iron (400 µM Fe-EDTA). S2, S5 and S6 denote transgenic *Arabidopsis* strains of *MxbHLH30*. (**B**) The content of Fe and Zn in all lines under stress. The scale represents 1 cm. Data were taken as the mean and standard error of three replicates. Asterisk denotes a difference from the control that is extremely significant (** *p* ≤ 0.01).

Pre-cultured T_3_ generation WT (wild type), UL (unloaded line) and *MxbHLH30* transgenic *Arabidopsis* were inoculated into 1/2 Murashige and Skoog (MS) medium with different Fe-EDTA concentrations (100 μM, 4 μM, 400 μM). After two weeks, the plants were phenotypically observed and sampled. The phenotype of the wild-type and UL transgenic strains and the *MxbHLH30* transgenic strains (S2, S5 and S6) were not significantly different under normal Fe concentration (100 μM). The results showed that the transgenic plants under Fe stress (4 μM, 400 μM) had longer roots, less pronounced greening of the leaves, and a significantly higher survival rate than WT and UL plants (Figure 4A). Under Fe-deficient conditions, the Fe content in roots and shoots of MxbHLH30-OE lines (S2, S5 and S6) was increased by approximately 50% compared with the control (Figure 4B). The iron content was slightly decreased in the transgenic lines under the excess iron environment.

**Figure 5:**
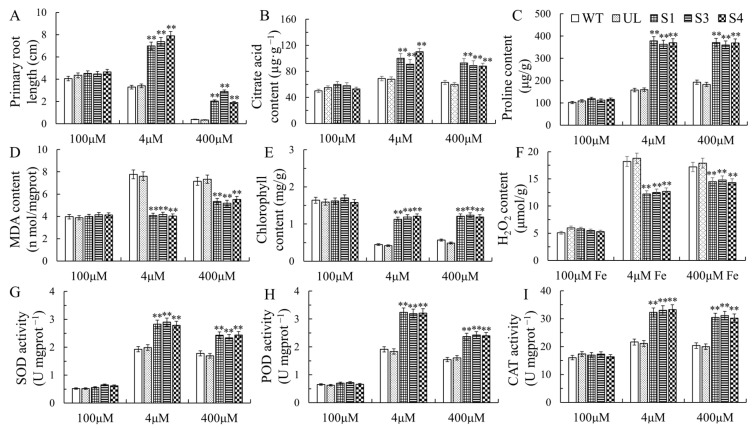
Relevant physiological indicators of *MxbHLH30* transgenic *Arabidopsis* plants under iron stress. These indicators were primary root length (**A**), citrate acid content (**B**), proline content (**C**), MDA (**D**), chlorophyll (**E**), H_2_O_2_ (**F**), SOD (**G**), POD (**H**) and CAT (**I**) enzyme activities. The average and standard deviation of three replicates were used as the data. Asterisk denotes a difference from the control that is extremely significant (** *p* ≤ 0.01).

The function of *MxbHLH30* response to iron deficiency or high-iron stress was clarified using the range of physiological indicators that were measured. The physiological indicators of all strains did not significantly differ under control conditions. Proline, iron, chlorophyll and the enzymatic activity of POD, CAT and SOD increased as a consequence of increased *MxbHLH30* expression under either high- or low-iron conditions, but MDA levels were lower than in the control group (Figure 5). The results of each index indicated that *MxbHLH30*-OE transgenic strains were more resistant to low- and high-iron stress than WT and UL transgenic lines.

Iron and zinc share the same transporter, and iron deficiency leads to excessive zinc uptake [21,32]. Several bHLH transcription factors have been found to be involved in plant Fe and Zn homeostasis [33]. The Zn content was significantly changed in the *MxbHLH30* transgenic plants (Figure 4B). To investigate whether *MxbHLH30* mediates Fe/Zn homeostasis to influence Fe content under Fe deficiency, we observed the Zn-dependent growth phenotype of transgenic plants. Overexpression of *MxbHLH30* under excess Zn stress (300 μM Zn) was also able to mitigate the deleterious effects on plant growth (Figure S3A). Excessive Zn stress resulted in the accumulation of large amounts of Zn in the roots of the MxbHLH30-OE lines, but there was no significant change in the shoots (Figure S3B). As in previous studies, excess Zn stress reduced Fe uptake in plants. Interestingly, the Fe content in shoots of MxbHLH30-OE lines was significantly higher than the WT and UL lines when exposed to excessive Zn. Compared with the control, the root–shoot ratio of Zn concentration rose in MxbHLH30-OE lines, while the root–shoot ratio of Fe concentration fell.

### 2.5. Expression Analysis of High- and/or Low-Iron-Stress-Resistant Downstream Genes in MxbHLH30-OE A. thaliana

**Figure 6:**
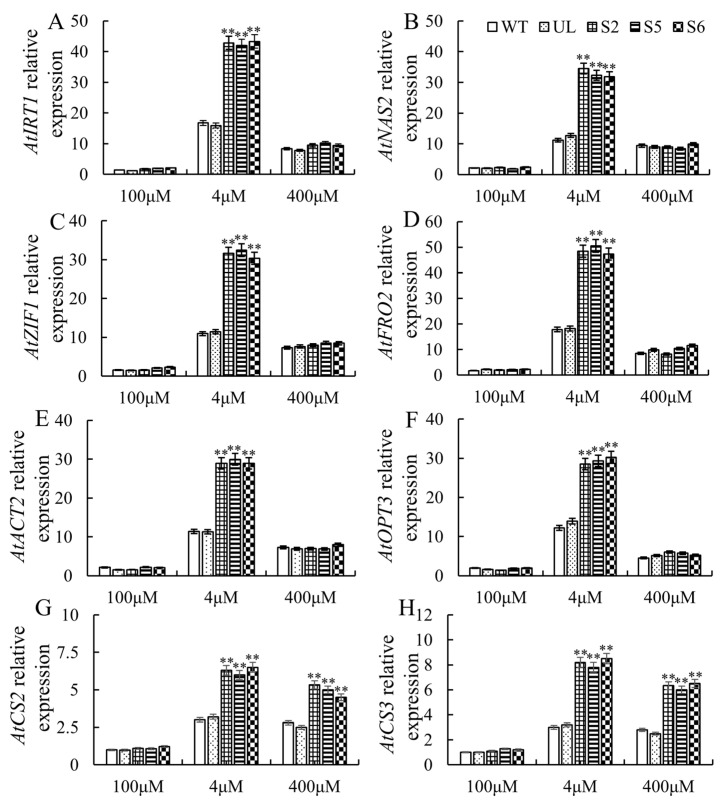
Expression analysis of Fe-stress-resistant downstream genes in WT, UL and transgenic *Arabidopsis.* Iron-stress-related genes are *AtIRT1* (**A**), *AtNAS2* (**B**), *AtZIF1* (**C**), *AtFRO2* (**D**), *AtACT2* (**E**), *AtOPT3* (**F**), *AtCS2* (**G**) and *AtCS3* (**H**). The average and standard deviation of three replicates were used as the data. Asterisk denotes a difference from the control that is extremely significant. (** *p* ≤ 0.01).

The joint action of iron transporter proteins, iron reductases and associated iron carrier genes is necessary for the molecular process of iron uptake in plants. The effective expression of these genes contributes to a greater level of plant iron stress tolerance. The control of iron homeostasis is greatly influenced by the bHLH transcription factor family of proteins. A number of significant related genes, including *AtNAS2* (Nicotianamine synthase)*, AtACT2* (Actin 2)*, AtZIF1* (Zinc-induced facilitator 1)*, AtIRT1* (ferrous iron transporter)*, AtFRO2* (Ferric reductase defective)*, AtOPT3* (Oligopetide Transporter) and *AtCS2*/*3* (citrate synthase), were examined in this experiment to determine how their transcript levels changed under iron treatment. As shown in Figure 6, the expression of these genes in all *Arabidopsis* lines (WT, UL, S2, S5 and S6) was up-regulated compared to the control (100 μM Fe-EDTA). Furthermore, the expression up-regulation of the downstream genes in the S2, S5 and S6 transgenic lines rose significantly following low-Fe-stress treatment. However, after high-Fe-stress treatment, the expression of six downstream genes in the S2, S5 and S6 transgenic lines, as well as the WT and UL lines, did not differ substantially, despite the expression of downstream-related genes being somewhat up-regulated in comparison to the control. Notably, the *AtCS2*/*3* was significantly upregulated in the S2, S5 and S6 transgenic lines after high-Fe-stress treatment (Figure 6). The aforementioned findings show that following iron stress treatment, iron stress resistance in plants is increased by the MxbHLH30 transcription factor, which positively regulates the expression of related genes.

## 3. Discussion

The bHLH TFs are considered to be the second most important transcription factors in plants. Since the first bHLH TF was identified in *Zea mays* L., bHLH TFs in plants have been continuously explored. *AtbHLH30* is essential for leaf morphogenesis and is associated with auxin signaling [34]. However, the relationship of bHLH30 to iron uptake and translocation is unknown. In this study, we found that the expression level of *MxbHLH30* was higher and the response to stress was faster than that of other MxbHLH after stress treatment (Figure S2), suggesting that *MxbHLH30* may be a major bHLH gene in *Malus xiaojinensis* in response to iron stresses. To analyze the correlation between *MxbHLH30* and iron stress, we validated the important role of *MxbHLH30* in response to abiotic stresses.

This study used homologous cloning to isolate the bHLH gene *MxbHLH30* from *Malus xiaojinensis*. The MxbHLH30 protein was localized to the nucleus (Figure 2). To further investigate the mechanism of MxbHLH30’s role in plant resistance to adversity, the identification of physiological and biochemical indicators in *Arabidopsis thaliana* as well as an examination of the expression of genes downstream of *MxbHLH30* in relation to iron stress were used. When subjected to stress, plants accumulate large amounts of reactive oxygen species (ROS) and induce oxidative stress, leading to membrane lipid peroxidation or the oxidation of biomolecules, where the degree of membrane lipid peroxidation is positively correlated with MDA content [35]. In addition, the amount of intracellular osmoregulatory chemicals like proline may increase and the amount of chlorophyll may decrease [36,37]. To reinforce the adaptive capacity of plants, environmental stress triggers antioxidant enzyme systems (protective enzymes such as POD, SOD, CAT, etc.) or produces a series of ROS-scavenging metabolites (ascorbic acid, glutathione, anthocyanin, etc.) to regulate the dynamic balance of ROS in vivo [38]. In our study, physiological and biochemical results showed that *MxbHLH30* overexpression reduced the severity of plant damage brought on by iron deficiency and iron overload while improving plant adaptability to adverse environments.

The promoters of *NAS4* (Nicotianamine synthase 4), *FRO3* (Ferric reductase defective 3) and *ZIF1* (Zinc-induced facilitator 1) can all be directly bound by the AtbHLH47 (PYE) protein, which is implicated in Fe homeostasis by suppressing the expression of these genes [39]. Kurt and Filiz [40] showed that heterodimerization of AtbHLH29 (FIT) with proteins encoded by bHLH transcription factor Ib subgroups bHLH38, bHLH39 or bHLH101 increases tolerance to iron deficiency. Comparable research has demonstrated that FIT activation upregulates *FRO2* and *IRT1/2* to enhance the absorption and transport of Zn and Fe to maintain homeostasis [41,42]. It is interesting to note that following extended exposure to high external Zn concentrations, the transcription factors FIT and bHLH Ib (bHLH38/39/100/101) triggered by iron deficiency showed significantly elevated transcript levels [25]. Expression of the metal tolerance proteins MTP3 and HMA3, which regulate Zn tolerance, is also partially dependent on FIT activity [33]. In addition, the knockdown of FBP (FIT-binding protein), which eliminates the DNA-binding ability of FIT, enhanced *NAS* gene expression involved in the regulation of iron and zinc homeostasis [43]. *IRT1* senses the concentration of non-Fe metals and coordinates its degradation to avoid toxicity caused by the accumulation of Zn and Mn [44]. *NAS*, the NA vesicular membrane transporter *ZIF1* and the phloem-specific iron transporter *OPT3* can also participate in metal uptake and heavy metal detoxification [45,46,47,48]. Following the application of low-iron stress, the expression levels of *AtNAS2, AtIRT1*, *AtZIF1*, *AtFRO2* and *AtOPT3* were noticeably more up-regulated in MxbHLH30-OE lines (Figure 6) and the Fe content rose significantly. Moreover, the root–shoot ratio of iron content in MxbHLH30-OE lines was reduced in the excess zinc environment, leading to the enrichment of scarce iron in the shoot (Figure S3B). It is shown that plants receive physiological signals and then may transduce them to downstream genes via *MxbHLH30*, allowing regulatory networks to respond to various stresses (such as Fe and Zn), and may fine-tune the expression of Fe stress-related genes in different tissues, balancing the homeostasis of Fe and other metal ions and improving plant resistance to low Fe stress.

It has been reported that iron stress induces the increased expression of *CS* genes and an increase in citrate and other carboxylates [22,49]. On the one hand, the high content of citrate acid helps plants’ uptake and long-distance transportation of Fe from the poor iron environment [50], and on the other hand, it promotes the chelation of redundant metal ions for detoxification [51,52]. Under high-iron stress, significant upregulation of *AtCS2/3* expression (Figure 6) and elevated citrate acid content were observed in *MxbHLH30* transgenic plants (Figure 5), which is consistent with previous studies [26,53]. This result suggests that the involvement of citrate in detoxification may confer on *MxbHLH30* transgenic plants a higher tolerance to high-iron stress.

**Figure 7:**
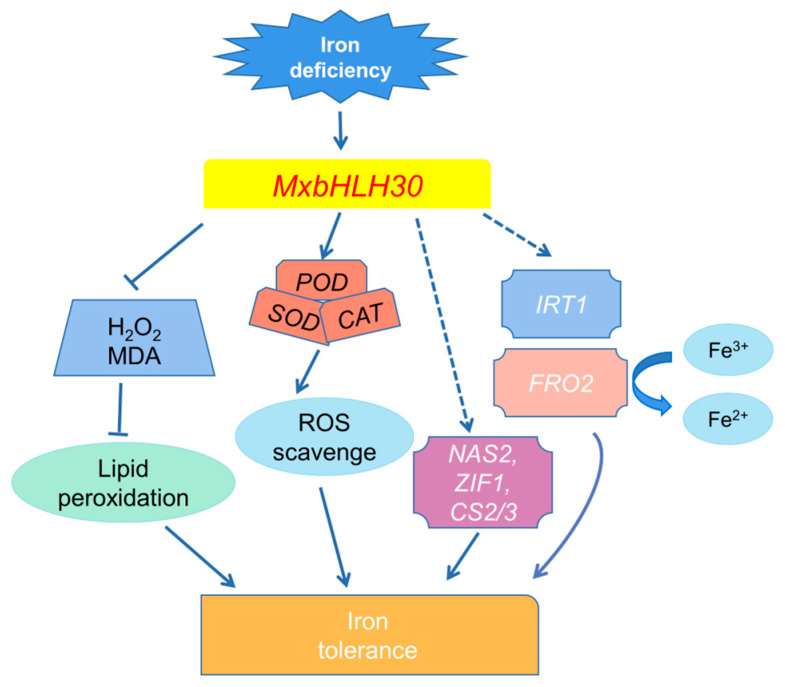
A possible model for the role of *MxbHLH30* during plant resistance to iron stress. The expression of *MxbHLH30* was induced by iron deficiency and iron excess. The activated *MxbHLH30* substantially reduced cellular damage by reactive oxygen species as well as lipid peroxides, increased the citric acid content and promoted the up-regulated expression of stress-related genes, thereby conferring iron uptake and utilization capacity in overexpressed *MxbHLH30* plants.

The above findings offer supportive evidence that *MxbHLH30* participates in growth regulation under iron stress. Thus, based on the advancement of prior investigations and the aforementioned experimental findings, we proposed a possible model for resolving the mechanism of action of *MxbHLH30* in response to iron stresses (Figure 7). Stimulation of the low-Fe environments induced a high expression of *MxbHLH30*, which enhanced the scavenging of reactive oxygen species by SOD, POD and CAT and reduced cell membrane lipid peroxidation. Reduced cellular damage contributed to significant up-regulation of a series of stress-responsive genes and functioned in their respective pathways, providing *MxbHLH30* transgenic plants with a higher capacity to modulate iron homeostasis under iron deficiency.

## 4. Materials and Methods

### 4.1. Plant Materials and Growth Conditions

*Malus xiaojinensis* histoculture seedlings pre-cultured for 40 days were inoculated onto 1/2 MS medium supplemented with 1.5 mg/L IBA for rooting culture, and vigorous inter-root growth was observed after about 45 days [54]. To allow further growth of the histopathic seedlings, they were transferred to Hoagland hydroponic solution to continue the culture. The culture solution was changed every 2–3 days. *Malus xiaojinensis* were cultured in a greenhouse at a temperature of 25 °C with a photoperiod of 16 h of light and 8 h of darkness. Then, when the plants developed to have 6–8 mature leaves, they were subjected to low-iron (4 µM Fe-EDTA Hoagland hydroponic solution), high-iron (400 µM Fe-EDTA Hoagland hydroponic solution), high-salt (200 mM NaCl) and low-temperature stress (4 °C). Culturing with normal Hoagland hydroponic solution (100 µM Fe-EDTA) was used as a control treatment. At 0, 1, 3, 6, 9, 12 and 24 h, fresh leaves and roots were harvested and kept at −80 °C [55].

### 4.2. Cloning and qPCR Analysis of MxbHLH30

Using The EasyPure Plant RNA Kit (TransGen Biotech, Beijing, China), the total RNA of *Malus xiaojinensis* was extracted under various circumstances. The first strand of the *Malus xiaojinensis* cDNA was created using the HiFiScript gDNA Removal cDNA Synthesis Kit (Kangweishiji, Beijing, China). Using the cDNA obtained above as a template, the whole length of *MxbHLH30* was amplified by polymerase chain reaction (PCR). Specific primers MxbHLH30-F and MxbHLH30-R, designed and synthesized according to the homologous sequence, were used for this procedure. Gel-purified DNA fragments were ligated into vectors, screened for positive clones, and sent for sequencing [56].

### 4.3. Subcellular Localization

The pSAT6-RFP-N1 vector was used to create an overexpression vector by cloning the open reading frame (ORF) of *MxbHLH30* between the *Xma* I and *Xbal* I sites. A recombinant plasmid containing the MxbHLH30-GFP with modified red-shifted GFP at the *Xma* I-*Xbal* I locus was introduced into the tobacco leaves for subcellular localization assays. Confocal microscopy (EVOS Floid, Seoul, Republic of Korea) allowed the transient expression of the MxbHLH30-GFP fusion protein to be seen.

### 4.4. qPCR Analysis

Expression of *MxbHLH30* in *Malus xiaojinensis* was determined by quantitative reverse transcription polymerase chain reaction (qRT-PCR) [57]. The actin primers (Actin-F; Actin-R, Table S1) were based on the GenBank database, whereas the primers, MxbHLH30-qF and MxbHLH30-qR (Table S1) were created based on the specific sequences of *MxbHLH30*. The thermal cycling procedure was 95 °C for 5 min, 95 °C for 5 s, 58 °C for 40 s, 72 °C for 15 s, and then 40 cycles starting from the second step, 72 °C for 5 min, 4 °C for storage. Using the 2^−ΔΔCT^ approach, it was possible to figure out the gene’s relative expression.

### 4.5. Overexpression of MxbHLH30 in A. thaliana

The *MxbHLH30* gene was inserted into the pCAMBIA2300 vector between the *Xma* I and *Xbal* I enzyme digestion sites to generate the overexpression vector pCAMBIA2300-MxbHLH30. This process required ligation by T_4_ DNA ligase. In order to genetically transform *Arabidopsis thaliana*, the constructed vector was used. *Arabidopsis thaliana* was genetically transformed using Agrobacterium-mediated inflorescence infestation [58], and in a medium of 1/2 MS with 50 mg/L kanamycin, transgenic lines were grown. Positive plants were screened with WT (wild-type) and UL (unloaded line, plant transformed with vectors not linked to target gene) as controls until no traits segregated in the offspring [53].

### 4.6. Determination of Related Physiological Indexes

Plant material from all treatments (WT, UL, S2, S5 and S6) was collected for determination. Xu et al.’s [59] method was used to calculate the amount of chlorophyll. The iron and zinc contents of the samples were determined using an inductively coupled plasma emission spectrometer (Agilent 5800 ICP-OES, Santa Clara, CA, USA), after acid and high-temperature digestion. The methods of Jiang et al. [60] and Liu et al. [28] were used to detect the contents of MDA and free proline. The Nitrotetrazolium Blue chloride (NBT) photoreduction method [61], guaiacol method [62] and UV absorption method [3] were used to measure the activity of enzymes including CAT, POD and SOD. At the same time, the hydrogen peroxide [63] and superoxide anion contents [64] were also determined.

### 4.7. Statistical Analysis

For the one-way ANOVA test, IBM SPSS Statistics 21 was employed. Five measurements of each indicator were made, and the results were presented as mean ± standard error (SE). Each experiment was performed three times each. Significant differences were used to describe statistical disparities. *, *p* ≤ 0.05, **, *p* ≤ 0.01.

## 5. Conclusions

In this study, we discovered the *Malus xiaojinensis MxbHLH30* transcription factor gene and demonstrated its nuclear localization. According to a number of physiological and biochemical indicators under iron stress, the overexpression of *MxbHLH30* promoted the scavenging of reactive oxygen species, altered plant redox homeostasis, increased the citric acid content and enhanced the resistance of transgenic *Arabidopsis* to iron deficiency and iron excess. Moreover, we also uncovered an aspect of crosstalk between iron homeostasis and zinc partitioning that is mediated by *MxbHLH30*. *MxbHLH30* may be functioning as a positive modulator of iron homeostasis, according to this study’s analysis of the molecular mechanisms behind the link between the *MxbHLH30* and plant resistance to abiotic stresses.

## Data Availability

Data will be made available on request.

## Supplementary Materials

The supporting information can be downloaded at: https://www.mdpi.com/article/10.3390/ijms26010368/s1.
