# Supplementary material for: Malus xiaojinensis MxbHLH30 Confers Iron Homeostasis Under Iron Deficiency in Arabidopsis
Source: Int J Mol Sci. 2025 Jan 3;26(1):368. doi: 10.3390/ijms26010368 (PMC11720179; doi:10.3390/ijms26010368)
Supplement: Supplementary file 1 [file ijms-26-00368-s001.zip › Legends of supplementary materials.pdf]

Legends of supplementary materials

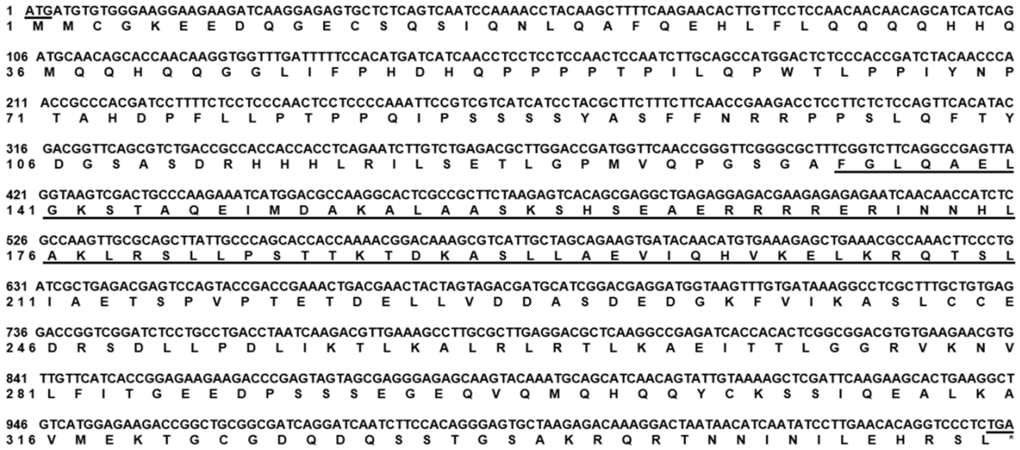

Figure S1. Nucleotide sequences of *MxbHLH30*. The region marked by the longer black underline is the conserved structural domain of bHLH.

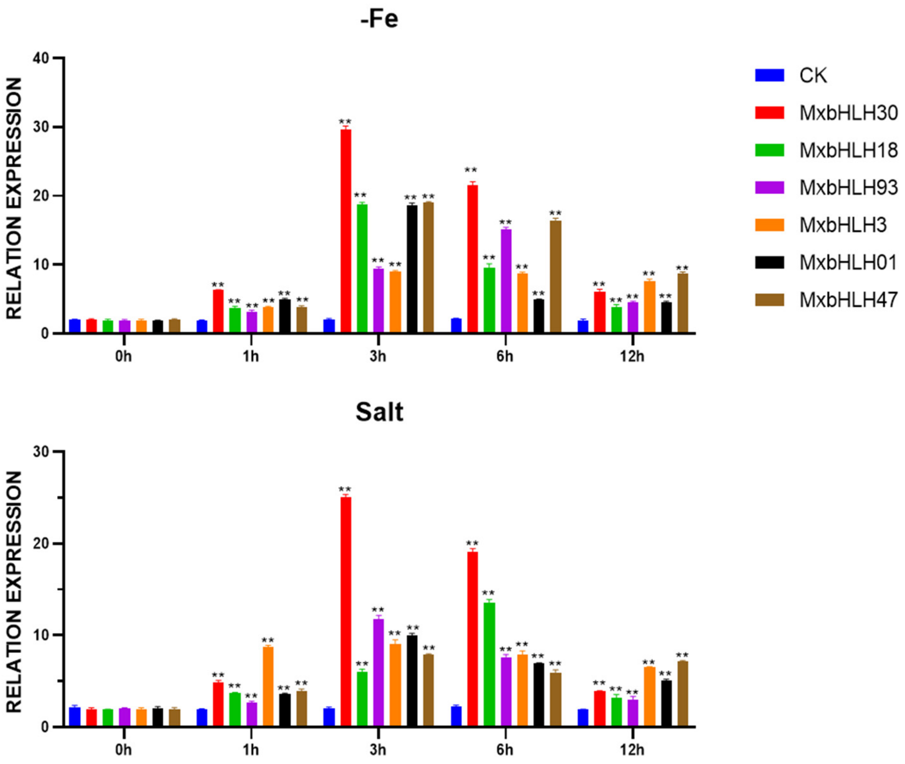

Figure S2. Relative expression of some bHLH genes significantly induced by iron and salt treatments (** $p \leq 0.01$).

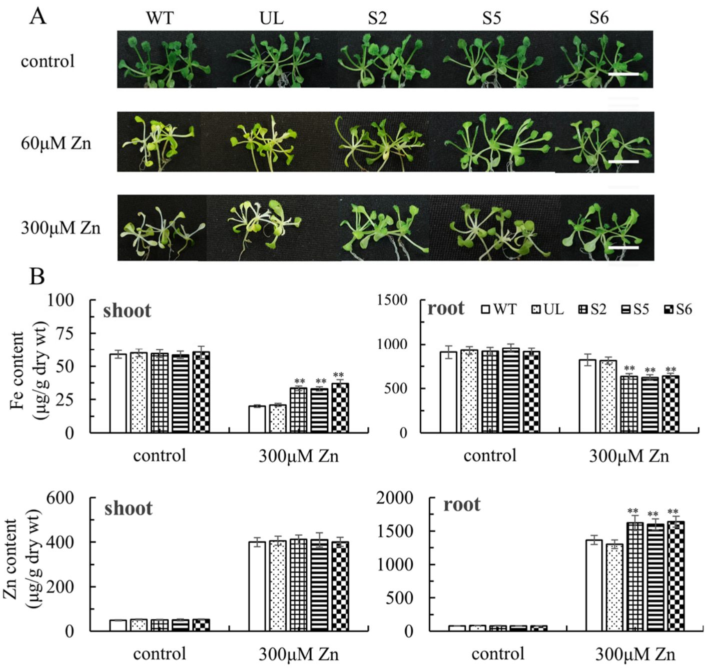

Figure S3. *MxbHLH30* mediated Zn/Iron homeostasis in *Arabidopsis*. (A) The phenotype of S2, S5, S6, WT, and UL under excess Zn. (B) The content of Fe and Zn in all lines under stress. The scale represents 1 cm. Data were taken as the mean and standard error of three replicates. Asterisk denotes a difference from the control that is extremely significant (** $p \leq 0.01$).
