# Supplementary material for: Malus xiaojinensis MxbHLH30 Confers Iron Homeostasis Under Iron Deficiency in Arabidopsis
Source: Int J Mol Sci. 2025 Jan 3;26(1):368. doi: 10.3390/ijms26010368 (PMC11720179; doi:10.3390/ijms26010368)
Supplement: Supplementary file 1 [file ijms-26-00368-s001.zip › Table S1.pdf]

Table S1. List of primers used in this study

Primer Name	Primer sequence (5'→3')	Function
MxbHLH30-F	ATGATGTGTGGGAAGGAAGA	Clone full length of *MxbHLH30*
MxbHLH30-R	TCAGAGGGACCTGTGTTC	
Actin-F	ACACGGGGAGGTAGTGACAA	q-PCR for *Actin*
Actin-R	CCTCCAATGGATCCTCGTTA	
MxbHLH30-qF	CACCAAAACGGACAAAGCGT	q-PCR for *MxbHLH30*
MxbHLH30-qR	GTACTGGACTCGTCTCAGCG	
AtCBF1-F	GGCCGTAAGAAGTTTCGTGA	q-PCR for *AtCBF1*
AtCBF1-R	ATCGTCTCCTCCATGTCCAG	
AtCBF2-F	AACTCCGGTAAGTGGGTGTG	q-PCR for *AtCBF2*
AtCBF2-R	CGGCGTATAAATAGCCTCCA	
AtCBF3-F	ACAGAGGAGTTCGTCGGAGA	q-PCR for *AtCBF3*
AtCBF3-R	ACCAACGTCTCCTCCATGTC	
AtCOR15a-F	GGCCACAAAGAAAGCTTCAG	q-PCR for *AtCOR15a*
AtCOR15a-R	CTTGTTTGCGGCTTCTTTTC	
AtCOR15b-F	CACAACGTAGGAGCAAGCA	q-PCR for *AtCOR15b*
AtCOR15b-R	GAGGATGTTGCCGTCACTTT	
AtKIN1-F	TGTCAGAGACCAACAAGAATGC	q-PCR for *AtKIN1*
AtKIN1-R	CCGCATCCGATACACTCTTT	
AtIRT1-F	GCCCCGCAAATGATGTTACC	q-PCR for *AtIRT1*
AtIRT1-R	TCCAATGACCACCGAGTGAA	
AtFRO2-F	ATCGAAAGTCGCCACACCAT	q-PCR for *AtFRO2*
AtFRO2-R	GAGCCACAAACATCGCCAAG	
AtNAS2-F	CGACGTGGTTAATTCGGTGG	q-PCR for *AtNAS2*
AtNAS2-R	CATAACCACACACCGTCCGA	
AtACT2-F	TGTGCCAATCTACGAGGGTTT	q-PCR for *AtACT2*
AtACT2-R	TTTCCCGCTCTGCTGTTGT	
AtZIF1-F	CGATATGCTGGGGCACTGA	q-PCR for *AtZIF1*
AtZIF1-R	CCGGTTATGGCAGACACACT	

AtOPT3-F	AAGCTTACTATAAACAGAGCCTTAGCTT	q-PCR for *AtOPT3*
AtOPT3-R	ACAGGATCAACAAGGTACCTCCTC	
